# Community structure and diversity characteristics of rhizosphere and root endophytic bacterial community in different *Acacia* species

**DOI:** 10.1371/journal.pone.0262909

**Published:** 2022-01-31

**Authors:** Zong-sheng Yuan, Fang Liu, Shi-bin He, Li-li Zhou, Hui Pan

**Affiliations:** 1 Institute of Oceanography, Minjiang University, Fuzhou, China; 2 College of Life Sciences, Fujian Agriculture and Forestry University, Fuzhou, China; 3 Zhangpu Zhongxi State-Owned Forest Yard, Zhangzhou, China; University of Salento: Universita del Salento, ITALY

## Abstract

Rhizosphere and endophytic microbiota significantly affect plant growth and development by influencing nutrient uptake and stress tolerance. Herein, root and rhizosphere soil of *Acacia* species were collected and analyzed to compare the structural differences of the rhizosphere and root endophytic bacterial communities. High-throughput 16S rRNA gene sequencing technology was employed to analyze the rhizosphere and root endophytic bacterial communities. A total of 4249 OTUs were identified following sequence analysis. The rhizosphere soil contained significantly more OTUs than the root soil. Principal component analysis (PCA) and hierarchical cluster analysis indicated that bacterial communities exhibited significant specificity in the rhizosphere and root soil of different *Acacia* species. The most dominant phylum in the rhizosphere soil was Acidobacteria, followed by Proteobacteria and Actinobacteria, whereas the dominant phylum in the root soil was Proteobacteria, followed by Actinobacteria and Acidobacteria. Among the various *Acacia* species, specific bacterial communities displayed different abundance. We systematically described the core bacteria in the rhizosphere and root endophytic bacterial communities and predicted their relevant functions. The type and abundance of specific bacteria were correlated with the nutrient absorption and metabolism of the *Acacia* species. This study addresses the complex host-microbe interactions and explores the rhizosphere and root bacterial community structure of different *Acacia* species. These results provide new insights into the role of rhizosphere and root endophytic bacterial communities on the growth and reproduction of *Acacia*, thus informing future efforts towards sustainable development and utilization of *Acacia*.

## Introduction

Plants are sophisticated organisms and harbor many diverse microorganisms either on their surface or inside their system. Plants and microorganisms co-evolve and interact in nature [1–4]. For plants, the microbiota is essential in their growth and development [3,5,6], stress resistance [7,8], nutrients absorption [9], and sustainable production [10,11]. For example, the rhizosphere soil microbial system adjusts the community structure to have enough resistance in a growing season to deal with moderate drought and enhance plant tolerance [12]. Bacterial microbiota improves nutrient transport and a significant effect on plant growth and yield [13,14]. The research on the interaction between the bacterial microbiota and plants is mainly to solve four basic questions: Who is there? what are they doing? Who is active there? What is the relationship between their activities and ecosystem functions? [12].

*Acacia* plants are evergreen trees belonging to the *Acacia* genus of the Mimosa family. *Acacia* species exhibit rapid growth, strong sprouting ability, well-developed root system, tolerance to barrenness, salinity, and drought. Thus, they are ideal tree Species for the reforestation of sandy soils in coastal areas [15]. Several studies have been conducted on *Acacia* introduction, cultivation techniques, and management [16,17]. In the study of the structure, diversity, and function of the *Acacia* microbiota, Ana thinks the impact of *Acacia* on the soil microbial community depends more on the litter characteristics of the tree species rather than the origin of the tree species [18]. Mixed plantations of *Eucalyptus urophylla × E*. *grandis* grandis and *Acacia* mangium improve soil quality and the corresponding microbial indicators show a strong positive correlation [19]. However, there are not many reports. The research on the structure, diversity, and function of microorganisms in the root and rhizosphere of *Acacia* will provide important scientific basis for soil improvement, soil fertility maintenance, water and soil conservation and other ecological benefits of *Acacia* plants.

Metagenomics, macrotranscriptomics and other technologies can more accurately understand the activities and physiological potential of plant-related underground microbiota [20,21]. In this study, Illumina NovaSeq high-throughput sequencing technology was employed to analyze and compare the diversity of rhizosphere and root endophytic bacterial communities in different *Acacia* species. The *Acacia* trees were planted at the Zhangpu Zhongxi State-Owned Forest Yard, Fujian, China, this is the southeast coast of China. *E*. *urophylla × E*. *grandis* trees planted in the same habitat were used as control. The function of the bacterial populations was predicted based on their classifications.

## Materials and methods

### Sample collection

Samples were collected from *Acacia* plantation at the Zhangpu Zhongxi State-Owned Forest Yard, Fujian, China. The region is of subtropical monsoon climate; The geographic coordinates are 117°56′E, 24°28′N; The annual average temperature is 21.8°C, the annual precipitation is 1600 mm. In November 2019, roots, rhizosphere soil, and non-rhizosphere soil samples were collected from five *Acacia* species: *Acacia crassicarpa*, A. Cunn. ex. Benth, *Acacia cincinnata* F. Muell., *Acacia melanoxylon* R.Br., *Acacia mangium* Willd., and *Acacia mearnsil* De Wild. Corresponding samples of *E*. *urophylla × E*. *grandis* trees in the same area were used as control (*E*. *urophylla × E*. *grandis* is the main cultivated tree species in the local environment). Of the above six tree species, each tree involved three samples, and each sample was randomly collected and repeated in 3 copies. A total of 54 samples were sequenced.

Sample collection: For each sample, trees were randomly selected. Root samples (50–60 cm in depth) were collected 1 m from the trunk. Soil attached to the root (diameter < 1 cm) was collected as rhizosphere soil. Soil collected 30 cm away from the plant roots was regarded as non-rhizosphere soil sample. All samples were put into a sterile sample bag immediately after collection and stored at -20°C within 24 hours. The root samples were subjected to three times ultrasonic surface cleaning and two times sodium chlorate surface disinfection treatments before the endophytic bacteria were detected. The internal tissues of the roots were selected for testing.

### Total DNA extraction

Total DNA was extracted from the plant and soil samples using Tiangen kit (Tiangen biotech co., LTD. Beijin, China) according to the manufacturer’s instructions. For the specific extraction process, please refer to Fang Liu’s method [22]. Total DNA concentration and purity were determined through Agarose gel electrophoresis. The DNA samples that passed the quality test were stored at −20°C for later use.

### 16S rRNA gene high-throughput sequencing

In preparation for sequencing, primers were designed according to the conserved regions. The bacterial 16S rRNA gene was amplified with a set of primers targeting the hypervariable V3–V4 region. The primers were V3F (5′-ACTCCTACGGGAGGCAGCAG-3′) and V4R (5′-TACNVGGGTATCTAATCC-3′) [22]. Sequencing adapters were added to the ends of the primers. PCR amplification was performed, and the products were purified, quantified, and homogenized to form a sequence library. The built library was first examined for quality. Libraries that passed quality control were sequenced using the Illumina NovaSeq sequencing platform. Libraires of small fragments were constructed using the paired-end sequencing mode (Beijing Novogene Co., Ltd.). OTUs were clustered by splicing and filtering Reads.

### Data processing

The OTU (Operational Taxonomic Units) with 97% similarity was selected to generate the expected dilution curve [23]. Biomarkers with statistical differences in different groups were identified using LEfSe (LDA Effect Size) [24]. Differences between or within groups were determined by examining sample distribution following NMDS (NonMetric MultiDimensional Scaling) analysis. The relationship between populations and environmental factors was determined using RDA (Redundancy analysis)/CCA (Canonical correspondence analysis) [25]. The differences in functions among similar samples or between sample types were calculated through KEGG metabolic pathway differential analysis using the PICRUSt (Phylogenetic Investigation of Communities by Reconstruction of Unobserved States) software [26]. Coexpression network analysis diagram was drawn using the space algorithm and python to show the abundance variation in each species in each sample.

## Results

### Alpha rarefaction curves and alpha diversity

High-throughput sequencing of the bacterial 16S rRNA gene (region *V5-V7*) was performed to explore bacterial microbiota diversity in the rhizosphere soil and roots of different *Acacia* species. Samples including the roots, rhizosphere soil, and non-rhizosphere soil were collected from various *Acacia* species and *E*. *urophylla × E*. *grandis* trees grown in the same area. The samples were coded and shown in Table 1.

**Table 1:** Codes related to the sample.

Species	Sample	Code	Species	Sample	Code
*A*. *crassicarpa*	root	HJ.A	*A cincinnata*	root	JJ.A
	rhizosphere soil	HJ.D		rhizosphere soil	JJ.D
	non-rhizosphere soil	HJ.E		non-rhizosphere soil	JJ.E
*A*. *melanoxylon*	root	HM.A	*A*. *mangium*	root	ZG.A
	rhizosphere soil	HM.D		rhizosphere soil	ZG.D
	non-rhizosphere soil	HM.E		non-rhizosphere soil	ZG.E
*A*. *mearnsil*	root	MZ.A	*E*.* urophylla × E*.* grandis*	root	AS.A
	rhizosphere soil	MZ.D		rhizosphere soil	AS.D
	non-rhizosphere soil	MZ.E		non-rhizosphere soil	AS.E

**Fig 1:**
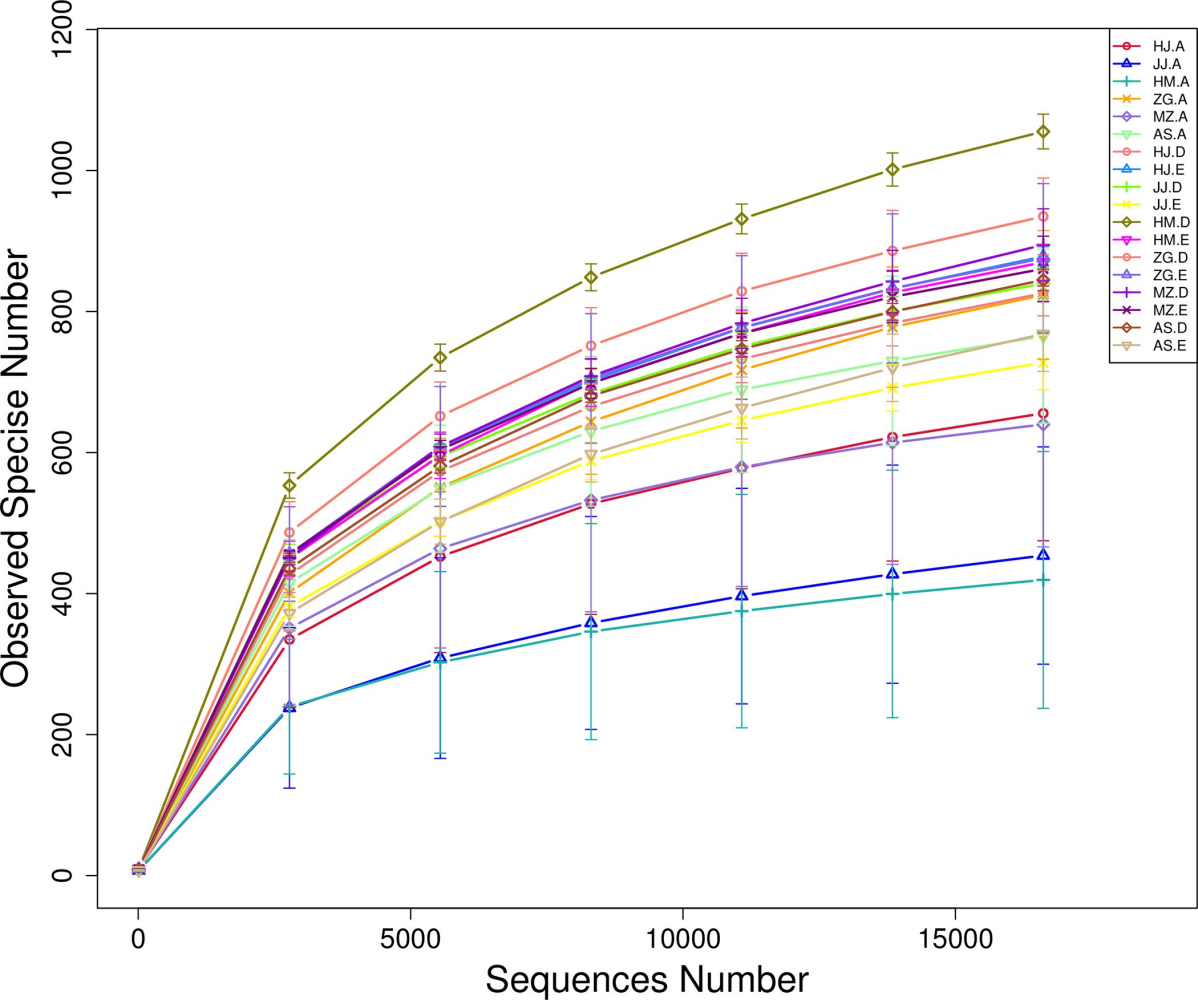
Average Good’s coverage estimates (%) of each plant compartment. Rarefaction curves were assembled showing the number of OTUs, defined at the 97% sequence similarity cut-off in mothur, relative to the number of total sequences (The code in the figure is shown in Table 1).

**Fig 2:**
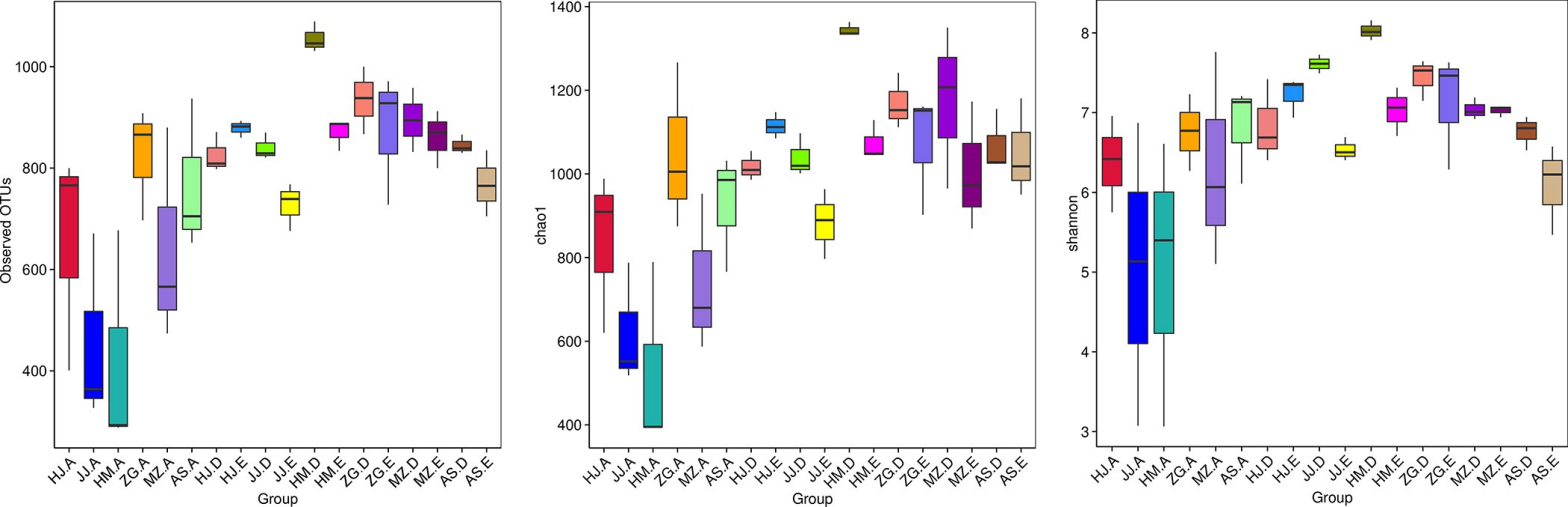
Alpha diversity estimates of the bacterial communities. a. Number of observed OTUs). b, Chao1 indices. c, Shannon diversity indices. Alpha diversity estimates represent 3 biological replicates for the rhizosphere soil, non-rhizosphere soil and root, were calculated in mothur with 10,000 iterations (The code in the figure is shown in Table 1).

Each sample has three biological replicates. A total of 54 samples were collected. High-throughput sequencing of the bacterial 16S rRNA genes generated 4,717,113 paired-ends sequences (Reads). In total, 4,230,732 high-quality sequences (clean tags) remained after read splicing and filtering. Each sample contained 61,029–90,690 clean tags. Using 97% as the similarity threshold, 4,269 bacterial OTUs were obtained from clustering. Dilution curve results showed that the diversity of rhizosphere bacterial communities was significantly higher than that of the root endophytic bacterial communities. Most of the root samples displayed peaks at 300–1000 OTUs, whereas the rhizosphere samples displayed peaks at around 1000 OTUs (Fig 1). Alpha diversity analysis also inferred similar OTU abundance (Fig 2). To further evaluate the sequencing depth, good’s coverages of all samples were calculated based on 10,000 iterative computations, which were highly comparable in the range of 98.0–99.6%. This indicated that the sequencing depth was sufficient to reliably describe the bacterial microbiota associated with plant roots and soil samples (These data were in NCBI GenBank, the relevant accession numbers were from SRR15701156 to SRR15701209).

**Fig 3:**
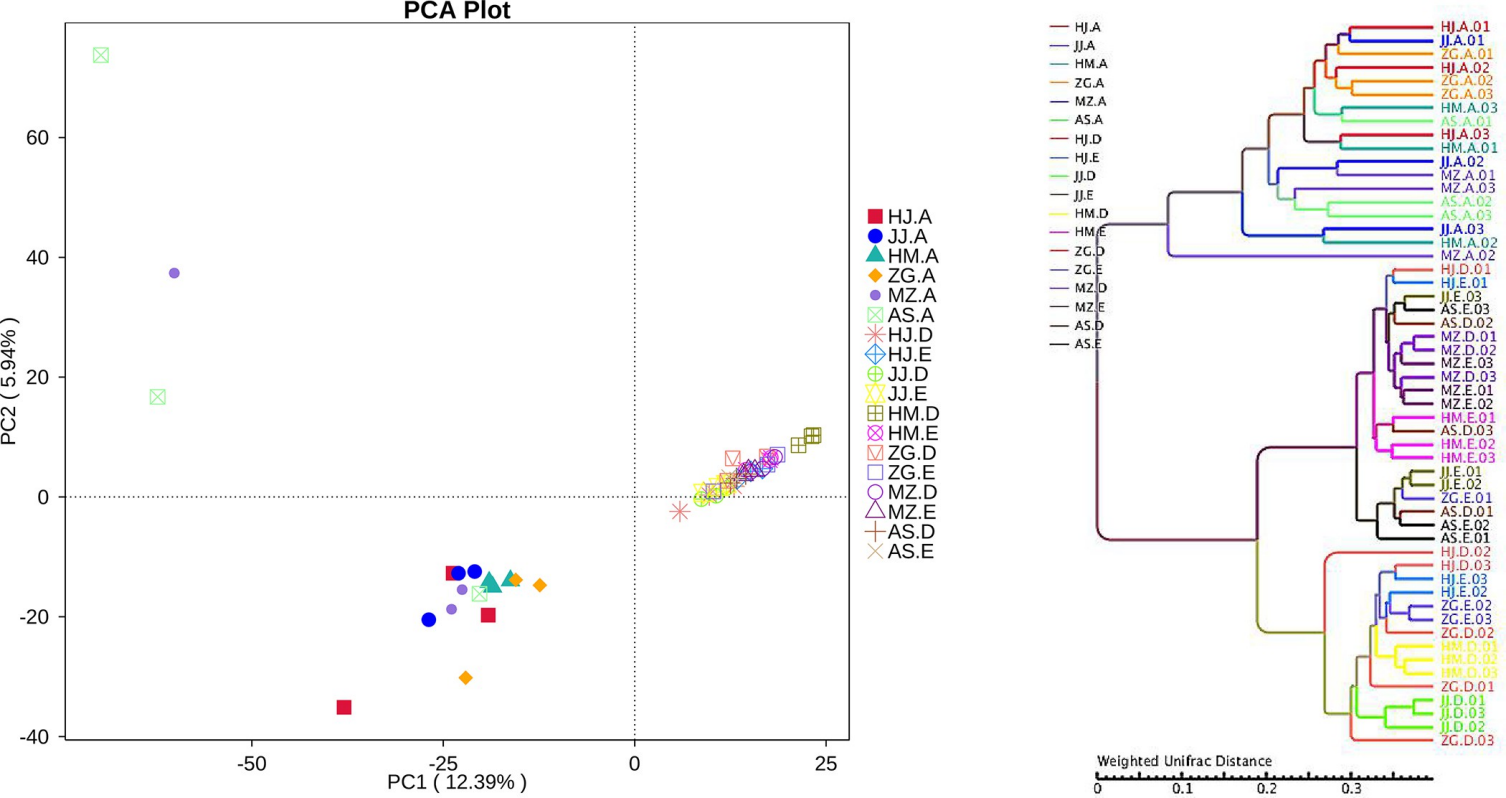
Plant compartment drives the composition of the bacterial communities at the OTU level. a, Principle component analysis (PCA) of square-root transformed samples based on rarefaction to 2000 reads per sample. b, Hierarchical clustering (group average linkage) of the samples based on Weighted Unifrac. PCA and hierarchical clusters were based on 3 biological replicates (rhizosphere soil, non-rhizosphere soil and root samples) and were constructed in PRIMER 7 with 10,000 iterations (The code in the figure is shown in Table 1; 01, 02, and 03 are biological repetitions).

Sequencing results from 54 samples were subjected to principal component analysis using Euclidean distance to compare the rhizosphere soil and bacterial community structure of root endophytes in different *Acacia* species and determine their main influencing factors. The principal components PC1 and PC2 represented 12.39% and 5.94% overlap, respectively, and the biological replicates in the same sample group were highly clustered (Fig 3A), indicating that the RNA-seq data had high repeatability and reliability. The close distance between the root samples of different *Acacia* species indicates that the root endophytic bacterial community composition is similar. The close distance between the rhizosphere and non-rhizosphere samples also indicates that the bacterial community composition of the rhizosphere and non-rhizosphere has a high degree of similarity. However, root samples are relatively discrete from rhizosphere and non-rhizosphere samples, indicating that there are differences in bacterial community composition between the two (Fig 3A). Additionally, hierarchical clustering (at the OTU and phylum levels) showed that the root samples, rhizosphere, and non-rhizosphere soil samples of different *Acacia* species were completely clustered. However, the *E*. *urophylla × E*. *grandis* root samples were more discrete (Fig 3B).

### Core bacteria microbiota within each plant compartment

**Fig 4:**
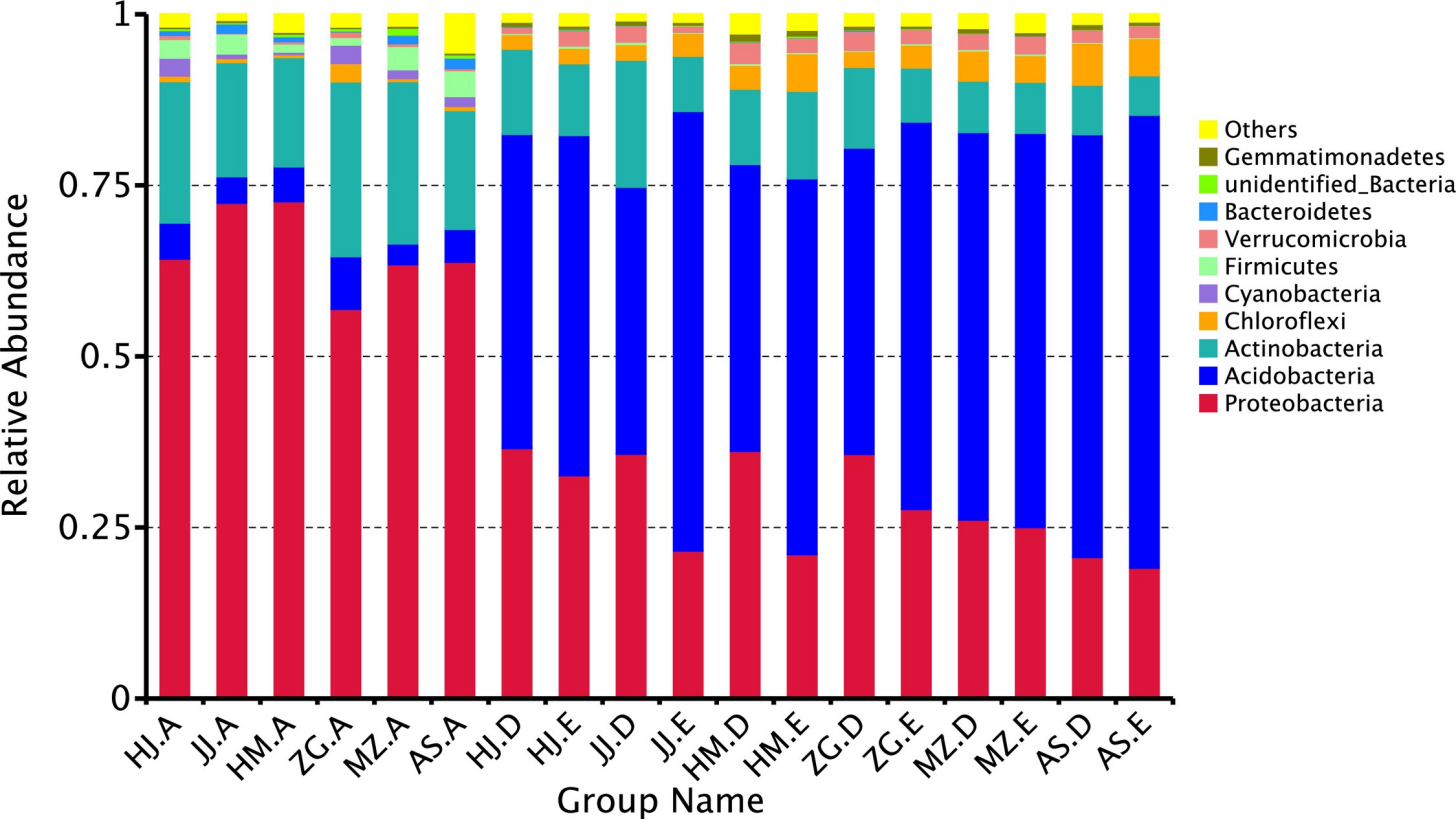
Dominant bacterial phyla detected in rhizosphere soil, non-rhizosphere soil and root compartments (the code in the figure is shown in Table 1).

The relative abundance of the rhizosphere and root endophytic bacterial communities at the phylum level were compared to further study the variations in the rhizosphere and root endophytic bacterial communities of different *Acacia* species. Through species annotation, Proteobacteria, Acidobacteria, and Actinobacteria were identified as the dominant phyla. By comparing the abundance ratio of the rhizosphere bacterial community to the root endophytic community in the studied trees, Proteobacteria, followed by Actinobacteria and Acidobacteria, were identified as the dominant bacteria in the root endophytic bacterial population (Fig 4). Regarding the bacterial community in the soil samples, Acidobacteria was the most abundant, followed by Proteobacteria and Actinobacteria. The abundance ratio of Proteobacteria and Actinobacteria in the root endophytic bacterial community was significantly higher than that in the soil samples. In contrast, the abundance ratio of Acidobacteria in the root endophytic bacterial community was significantly lower than that in the soil samples. Significant differences were found between the bacterial communities in the soil and root samples. Also, the abundance ratios of Acidobacteria and Chloroflexi in the root endophytic bacteria of *A*. *mangium* were remarkably higher than those of *E*. *urophylla × E*. *grandis* and other *Acacia* species. The abundance ratios of Firmicutes and Bacteroidetes in the endophytic bacteria of *A*. *melanoxylon* and *A*. *mangium* were substantially lower than *A*. *crassicarpa*, *A*. *cincinnata*, *A*. *mearnsil* and *E*. *urophylla × E*. *grandis*. Among them, *A*. *mangium* had the lowest abundance ratio of Bacteroidetes. The abundance ratios of Cyanobacteria in the endophytic bacterial communities of *A*. *crassicarpa* and *A*. *mangium* were significantly higher than that of other *Acacia* species and *E*. *urophylla × E*. *grandis*. Among the bacterial communities in the rhizosphere soil samples of the studied tree species, the abundance ratios of Proteobacteria and Actinobacteria were higher than that in the non-rhizosphere soil samples. In the non-rhizosphere bacterial communities, the abundance ratio of Acidobacteria was higher than that of the rhizosphere soil samples of the same tree species. The abundance ratios of Chlaroflexi and Verrucomicrobia in the bacterial communities of the soil samples were significantly higher than in the roots. The abundance ratio of Firmicutes in the root endophytic bacterial communities of the studied tree species was significantly higher than the abundance ratio of the same bacteria in the soil samples. These results show that the composition of the bacterial communities in different *Acacia* species is significantly different.

**Fig 5:**
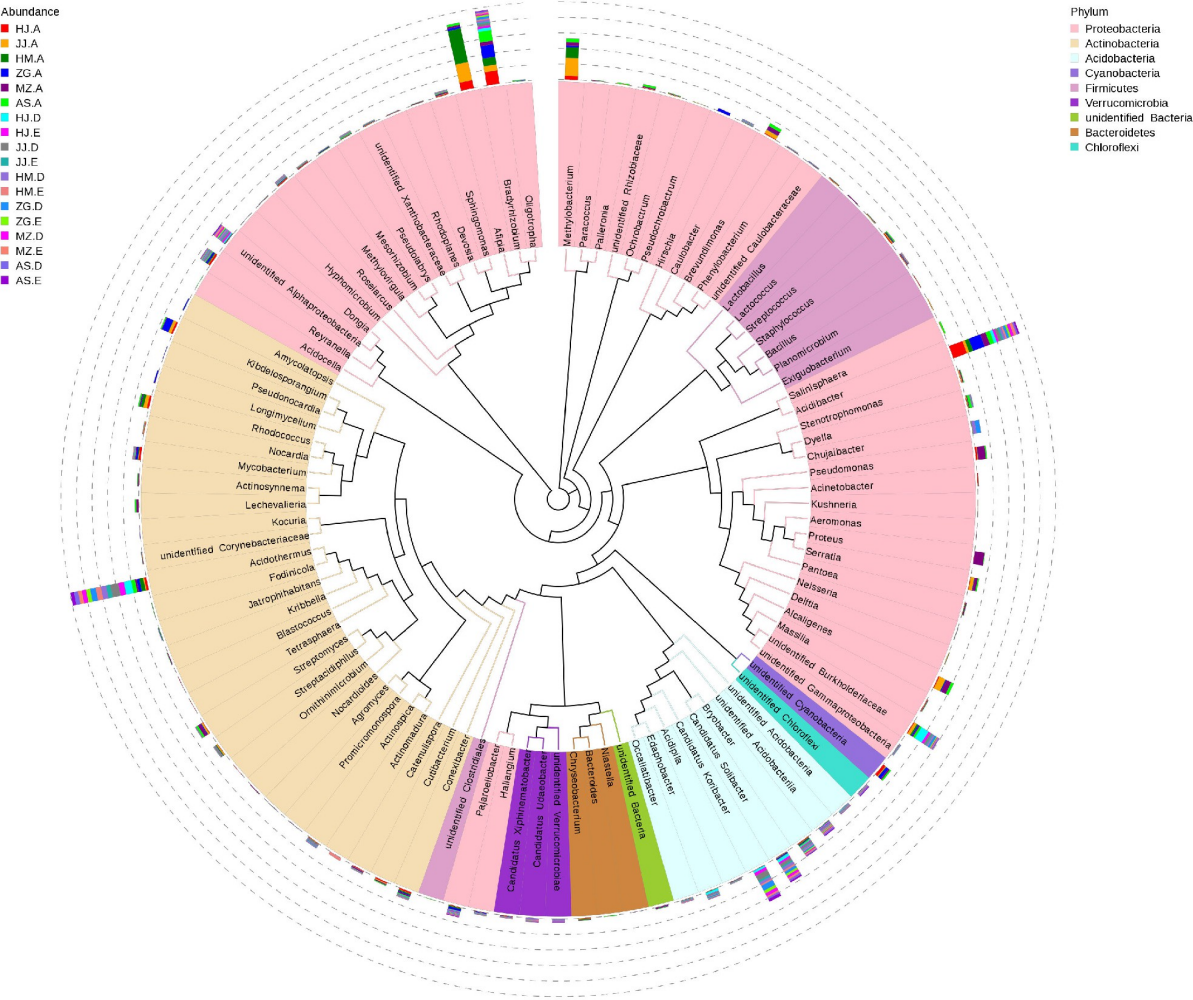
Top OTU members of the bacterial microbiome associated with the plant niches. Taxonomic dendrogram showing the core bacterial microbiome of each plant compartment. Color ranges identify genera within the tree (The code in the figure is shown in Table 1).

To further analyze the variation in bacterial communities in the rhizosphere soil and roots of different *Acacia* species, we studied the phylogenetic relationships of species at the genus level. Representative sequences of the top 100 genera were obtained through multiple sequence alignments. The abundance levels of Afipia, Methylobacterium, Brevundimonas, Rhodococcus, Streptomyces, and Alcaligenes in the root endophytic bacterial communities of different *Acacia* species were relatively high, while they were extremely low in the soil samples (Fig 5). Alphaproteobacteria, Koribacter, Solibacter, Bryobacter, and Acidobacteriia, were significantly higher in the soil samples than in the root tissue samples. Bradyrhizobium, Acidibacter, and Acidothermus accounted for a certain percentage in the soil and root tissue samples of all the studied species.

### PICRUSt analysis

**Fig 6:**
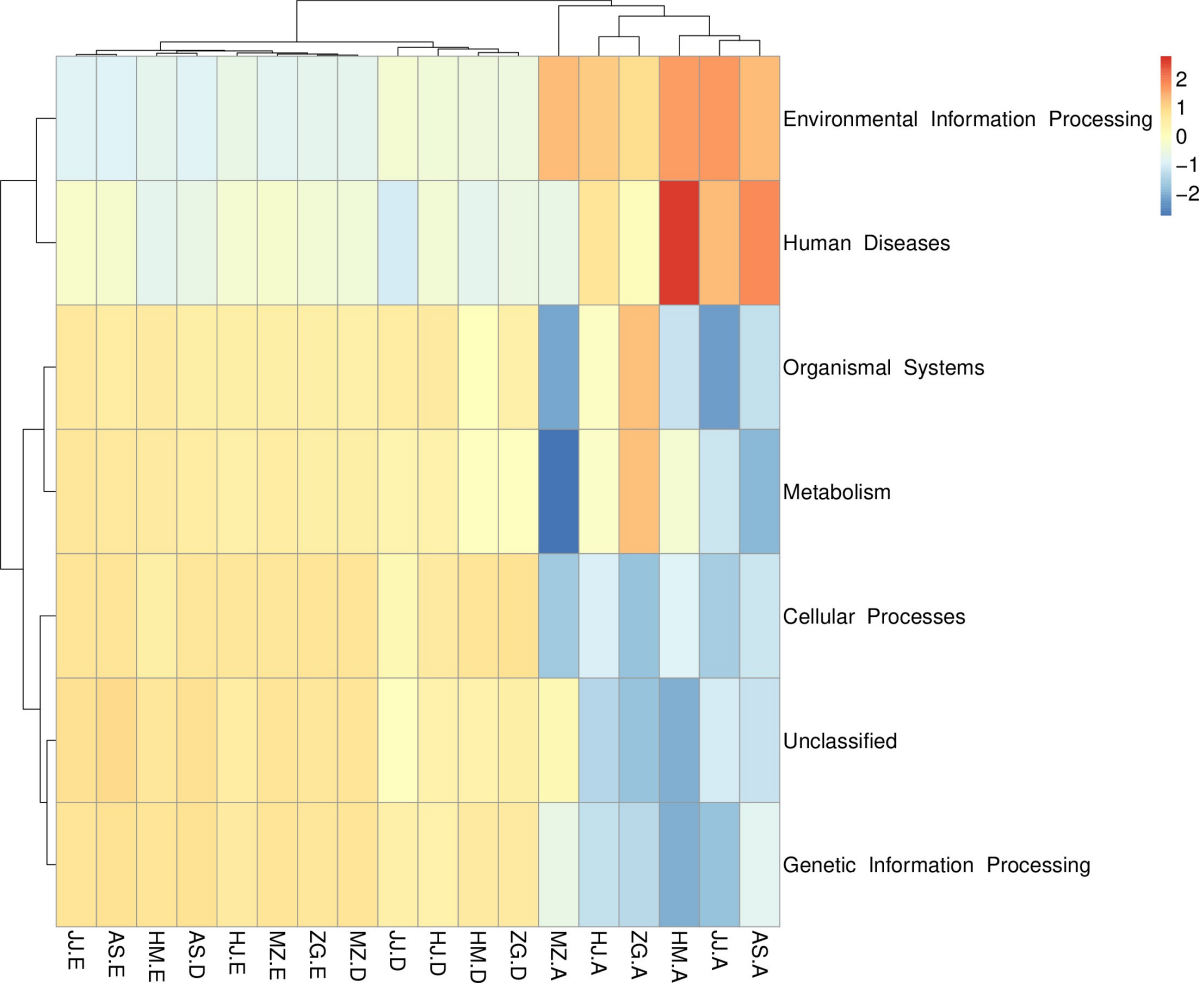
PICRUSt analysis of the bacterial microbiome in each plant compartment. The top 35 the bacterial microbiome and their abundance information in each sample were mapped and clustered from the functional difference level (The code in the figure is shown in Table 1).

To analyze the functions of specific bacterial communities, PICRUSt was used to conduct meta-genome function prediction. A heat map was drawn based on the abundance level in each sample and the function of the top 35 bacteria phyla in terms of the abundance. These bacteria were also clustered based on functional differences. The microorganisms in the soil samples participated in metabolism, cell biological processes, tissue systems, and genetic information processes. In comparison, the microorganisms in the root tissues were involved in environmental information processes (Fig 6). which showed significant difference in the functions of the soil microbes.

## Discussion

In this study, a comparison of the dilution curves of the root versus soil samples of different tree species indicated that the bacterial communities in the soil samples exhibited a higher degree of abundance. The main reason may be that *Acacia* is a legume nitrogen-fixing tree [27], and the resulting increase in nitrogen availability may be the most important factor in regulating soil microbial communities [19], so that nutrients derived from root secretions and mucus attract numerous rhizosphere organisms from the environment. However, plant-related bacteria must be highly competitive to succeed in the root of specific colonization [28–31]. Roots of different species vary in their ability to absorb and secrete nutrients, resulting in different bacterial communities colonized in the roots [32–37]. In this study, the rhizosphere soil and root interior of different *Acacia* tree species have different bacterial communities, indicating that deposition of root-like structures and exudates from host plant roots promotes chemical receivability of roots to colonize bacteria, resulting in a unique, highly abundant, and diverse microbial population [28]. The non-leguminous *E*. *urophylla × E*. *grandis* root samples have the most discrete clusters, indicating that the types of bacterial communities in their roots are different from *Acacia* samples, and that *E*. *urophylla × E*. *grandis* roots absorb and secrete nutrients differently from *Acacia*.

Herein, the dominant rhizosphere bacteria were Proteobacteria, Acidobacteria, and Actinobacteria (Fig 4). This was consistent with the results previously reported on *Acacia* [38], *Arabidopsis thaliana* [39], corn [40], rice [41], suggesting that the establishment of the rhizosphere bacterial community in *Acacia* followed the general law of the establishment of microbial communities. In our study, Proteobacteria and Acidobacteria were the dominant bacteria in the rhizosphere bacterial community of Acacia species on the southeastern coast of China, followed by Actinobacteria. The proportion of Proteobacteria and Acidobacteria in rhizosphere bacterial communities is an indicator of soil nutrient content. Proteobacteria are associated with nutrient-rich soil, it may play a key role in the establishment of bacterial communities in the soil, and the abundance of this phylum is higher in soil samples of legume trees [27]. whereas Acidobacteria is a K-strategist, grows slowly and has drought tolerance, and has a remarkable ability to grow in a nutrient-poor environment [42–44]. The results of the study indicate that dominant rhizosphere bacteria are ubiquitous or specific around the roots of certain plant species. This shows that we can "manage" the soil bacterial community by choosing to cultivate certain trees or plants, thereby changing and improving the soil nutrients and structure of the plantation [12], which has a good potential effect on the plantation.

The soil samples used in this study came from the coast of southeastern China, which belongs to the subtropical monsoon climate. The rainfall is abundant, leading to rapid nutrient loss, and the soil is barren and acidic. However, this study found the high abundance of bacterial communities in the rhizosphere soil of *Acacia*, indicating that *Acacia* can promote nitrogen input, improve soil ecology and enhance biogeochemical cycles [38]. This study also found that different *Acacia* tree species have different root endophytic bacterial dominant communities, and it has a low correlation with their corresponding rhizosphere soil bacterial dominant communities. For example, the abundance ratio of Acidobacteria and Chloroflexi in the roots of A. mangium is significantly higher than that of *E*. *urophylla × E*. *grandis* and other *Acacia* species. Among the rhizosphere soil samples, the abundance ratio of Acidobacteria and Chloroflexi of the A. mangium sample is the lowest. Variations in the abundance of these bacterial phyla in different *Acacia* species may influence the nutrition and energy intake of various *Acacia* species, especially nutrient intake from the soil. So, a more detailed study on the structure of these microbial communities and their role in forest ecosystems.

## Conclusions

This study collected and analyzed the root, rhizosphere, and non-rhizosphere soils of *Acacia* plants, compare the differences in the bacterial community structure in the rhizosphere and roots of different *Acacia* species, the core bacteria in the rhizosphere and root endophytic bacterial communities were identified, and their related functions were predicted. It is determined that the type and abundance of specific bacteria are related to different *Acacia* species. Our research provides new insights for understanding the role of rhizosphere and root endophytic bacterial communities on the growth and reproduction of *Acacia* and provides more basis for improving the soil nutrients and structure of plantations, and *Acacia* Sustainable development and utilization.
